# Hand Gesture Recognition Based on High-Density Myoelectricity in Forearm Flexors in Humans

**DOI:** 10.3390/s24123970

**Published:** 2024-06-19

**Authors:** Xiaoling Chen, Huaigang Yang, Dong Zhang, Xinfeng Hu, Ping Xie

**Affiliations:** 1 Institute of Electric Engineering, Yanshan University, Qinhuangdao 066004, China; xlchen@ysu.edu.cn (X.C.); yhg622822@163.com (H.Y.); zhangdong_0328@163.com (D.Z.); 17638352137@163.com (X.H.); 2 Key Laboratory of Measurement Technology and Instrumentation of Hebei Province, Institute of Electric Engineering, Yanshan University, Qinhuangdao 066004, China

**Keywords:** high-density surface electromyography (HD-sEMG), gesture recognition, feature selection, machine learning

## Abstract

Electromyography-based gesture recognition has become a challenging problem in the decoding of fine hand movements. Recent research has focused on improving the accuracy of gesture recognition by increasing the complexity of network models. However, training a complex model necessitates a significant amount of data, thereby escalating both user burden and computational costs. Moreover, owing to the considerable variability of surface electromyography (sEMG) signals across different users, conventional machine learning approaches reliant on a single feature fail to meet the demand for precise gesture recognition tailored to individual users. Therefore, to solve the problems of large computational cost and poor cross-user pattern recognition performance, we propose a feature selection method that combines mutual information, principal component analysis and the Pearson correlation coefficient (MPP). This method can filter out the optimal subset of features that match a specific user while combining with an SVM classifier to accurately and efficiently recognize the user’s gesture movements. To validate the effectiveness of the above method, we designed an experiment including five gesture actions. The experimental results show that compared to the classification accuracy obtained using a single feature, we achieved an improvement of about 5% with the optimally selected feature as the input to any of the classifiers. This study provides an effective guarantee for user-specific fine hand movement decoding based on sEMG signals.

## 1. Introduction

Human–machine interfaces (HMIs) [1,2,3] are widely used to assist disabled people in sports rehabilitation [4], prosthetics control [5,6], human–computer interaction [7,8] and with other forms of interactive equipment. The use of sEMG signals, as one non-invasive technology, plays an important role in HMIs, especially for some refined gesture recognition [9]. The sEMG signals used in most studies are sparse multi-channel sEMG signals obtained using multiple independent electrodes on a specific muscle. Although it can present high recognition accuracy [10], the collection of sEMG data requires quite precise positioning of the electrodes [2]. In contrast, the HD-sEMG [11,12,13,14] signals collected by two-dimensional electrode grids can provide spatial and temporal information, and the increased electrode density can enrich the information on muscle contraction, helping to enhance the performance of gesture recognition [15,16]. Therefore, HD-sEMG is excellent for exploring recognition and has the potential to overcome the above shortcomings [17].

In recent decades, many gesture recognition methods based on sEMG signals have been proposed [18,19,20]. The current methods can be summarized into two categories. One is called the traditional machine learning (ML) method based on manual features, such as the linear discriminant analysis classifier (LDA) [21], the support vector machine (SVM) [9,22], the Gaussian mixture model (GMM) [23,24], the hidden Markov model (HMM) [25] and others. Chen et al. used the K-nearest neighbor method (KNN), LDA and the quadratic discriminant analysis algorithm (QDA) and integrated an SVM to recognize different gestures with a recognition rate of 97.93% [26]. Firas et al. used the general regression neural network (GRNN) and Sym4 wavelet energy features to recognize eight kinds of hand movements and achieved a 95% recognition rate [27]. Another approach is the end-to-end deep learning (DL) method, such as the convolutional neural network (CNN) and long short-term memory (LSTM). Muhammad et al. built a simple convolutional neural network model and trained it on six gestures to reach 94.85% recognition [28]. Geng et al. proposed the GentNet convolutional neural network model to identify gestures in Capgmyo and CSL_HDEMG high-density sEMG data and achieved 99.5% and 89.3% accuracy, respectively [29]. Compared with traditional ML methods, DL-based myoelectric recognition has better accuracy and generalization ability in multiple classification problems. However, in actual system development, a real-time myoelectric control system based on deep learning is still difficult to achieve. To improve recognition accuracy, complex deep learning network models require a large amount of data to train them, which puts a heavy training burden on the user. In other words, the DL network requires a large number of samples and a lot of time for support training; it has certain difficulties in meeting the real-time requirements of pattern recognition. Therefore, ML-based gesture recognition still has great research value.

Additionally, good features also have a huge impact on improving recognition. Various features have been identified in the time domain, the frequency domain and the time–frequency domain [30,31]. Angkoon et al. proposed 37 time-domain and frequency-domain features and studied their classification performance. They then also evaluated 50 time-domain and frequency-domain features to improve the robustness of sEMG pattern recognition [32]. Early studies on sEMG gesture recognition often extracted a single feature as the input of the classifier, but because of the individual differences in sEMG signals, recognition varies greatly among different users. Therefore, multi-features have been used to improve the accuracy of gesture/motion classification. For example, Atzori et al. evaluated multiple sEMG features on three subdatasets of the Ninapro dataset. Finally, the combination of seven kinds of features obtained the highest gesture recognition accuracy of 75.32% [14]. Doswald et al. added four kinds of sEMG features based on the feature set proposed by Phinyomark et al. [32] to obtain an extended feature set, which obtained 97.29% accuracy in recognizing five kinds of gesture actions [33]. However, the above methods merely increased the number of feature types to improve gesture recognition performance and did not propose an effective feature selection algorithm. As the number of features increases, not only does the computational cost increase but the redundant features will also affect the pattern recognition performance. Meanwhile, due to the large variability of sEMG signals among different users, the recognition performance of a single feature varies greatly in cross-user gesture recognition.

Therefore, to address the challenges of increasing computational costs and limited cross-user robustness resulting from high feature dimensionality, it is necessary to use feature engineering to reduce the dimensionality of constructed high-dimensional feature sets. Feature dimensionality reduction can improve the accuracy and real-time performance of pattern recognition. Dimensionality reduction strategies are mainly divided into two categories: feature selection and feature projection. There are three commonly used feature selection methods: the filtering method, the wrapper method and the embedded feature selection model. Among them, the filtering approach utilizes the specific properties of the sEMG feature set itself to generate the target subset. The method is independent of the constructed pattern recognition model, which eliminates the tedious steps of training and testing the model. Therefore, filtered methods are often used for sEMG feature selection for high-dimensional data due to their versatility and efficiency. Yan et al. proposed an EMG-based feature classification method based on mutual information (MI) [34]. Firstly, the energy features of each sub-band of the wavelet packet transform (WPT) are utilized to construct an initial full feature set. Subsequently, MI theory is used to obtain a reduced feature set without affecting the classification accuracy. Khushaba et al. proposed an algorithm that combines MI and principal component analysis (PCA), denoted as mutual component analysis (MCA) [35]. The proposed MCA algorithm extends PCA by cropping noisy and redundant features before projecting the data. Subsequent use of PCA reduces the feature dimensionality and improves the recognition efficiency. Angari et al. compared two selection methods [36]. One was distance-based feature selection (DFSS), which uses the Mahalanobis distance between classes to determine a separability index. The second method was correlation-based feature selection (CFSS), which measures the amount of mutual information between features and classes. Finally, it was found that the CFSS method always uses fewer feature channels compared to the DFSS method. However, the above methods only consider the correlation between sEMG features and hand movement labels, filtering out a subset of features that have low relevance to a particular user. These methods do not consider that features with strong correlations contain duplicated categorical information between them. This duplication of information may lead to inefficient pattern recognition results.

In this study, we propose a feature selection method that combines mutual information, the Pearson correlation coefficient and principal component analysis (MPP). This method combines feature selection and feature projection techniques to efficiently filter the optimal subset of features that match a particular user. The MPP method mainly consists of two feature selection stages and one feature projection. Firstly, we use MI to make feature selections on the constructed original feature set to remove features with low relevance to the user. Secondly, we use the Pearson correlation coefficient to select features again, eliminating features with strong correlations and reducing repeated redundant features. Finally, we use PCA to reduce the dimensionality of the filtered feature subset to reduce the computational cost of pattern recognition and improve the efficiency of pattern recognition. The major contributions of the current work are as follows.

(1) In this study, a new MPP feature selection method is proposed. This method introduces the Pearson correlation coefficient in contrast to previous feature selection methods. After filtering out the subset of features that have low relevance to a particular user using the MI method, the Pearson correlation coefficient is used again for feature selection to filter out the strong correlation features that contain repetitive information. Finally, PCA is used to reduce the feature dimensions and improve the performance of pattern recognition.
(2) The final classification performance is not only related to the feature selection algorithm and data processing techniques but also to the design of the classifier. By comparing the classification performance of different classifiers, SVM combined with the MPP feature selection algorithm achieves satisfactory results under the conditions of real time and robustness. The combination of the MPP feature selection method with the machine learning classification model greatly reduces the computational cost and user burden. It provides a reference for future development of online gesture recognition systems.
(3) By using the MPP feature selection method proposed in this study, the optimal subset of features can be filtered for different users simply and efficiently. This method provides a practical approach to solving the problem of low cross-user pattern recognition performance.

## 2. Materials and Methods

### 2.1. Subjects

Fourteen healthy right-handed volunteers (nine men and five women, aged 20–26 years) were recruited for this study. They had no mental or cognitive impairments and had no previous experience of similar gesture recognition experiments. This study was approved by the Ethics Review Board of the First Hospital of QinHuagDao (2021A032). Informed and written consent was obtained from all subjects before any procedure of the experiments was performed.

### 2.2. Experimental Paradigm

**Figure 1:**
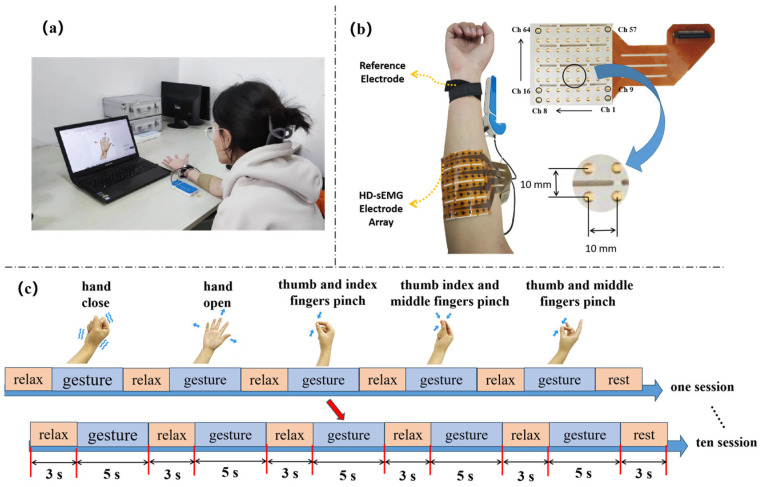
sEMG signals collected by a 64-channel high-density electrode array placed on the forearm. (**a**) Schematic diagram of HD-sEMG acquisition system. (**b**) Acquisition of electromyographic signals from forearm muscle groups. (**c**) Experimental paradigm flow chart.

Before the experiment, the subjects were asked to sit in a comfortable chair, put their arms on a table and remain relaxed (Figure 1a). They completed the appropriate gesture tasks based on information provided by the computer in front of them. Based on the frequency and usage of grasping in daily life, we chose the following five gesture movements: hand closed (HC); hand open (HO); thumb and index fingers pinched (TI); thumb, index and middle fingers pinched (TIM); and thumb and middle fingers pinched (TM) (Figure 1b) [37]. During the experiment, the subjects began preparing for the tasks while hearing sounds on the computer screen. All subjects were asked to perform twenty sessions with different gestures in turn. In each session, the subjects first remained in a relaxed state for 3 s, performed a hand gesture task for 5 s (from a relaxed state to the end of the corresponding gesture) and then returned to a relaxed state for 3 s before starting the next gesture task (Figure 1c).

### 2.3. Data Recordings

Before collecting data, the subjects needed to wipe their skin with 75% alcohol to reduce impedance. An 8 × 8 electrode array (64 channels) was placed along the ulnar flexor, digital flexor, flexor carpi radialis and palmar longus. The sEMG signals were acquired using the sessantaquattro System (OT Bioelettronica, Turin, Italy), as shown in Figure 1. The system mainly consists of a reference electrode, a 64-channel high-density electrode slice and an sEMG acquisition system. The electrode array consists of 64 gel elliptic electrodes, the center distance between the two adjacent electrodes is 10 mm and the reference electrode is fixed on the wrist. Here, we recorded HD-sEMG signals at a sampling rate of 2000 Hz with a 16-bit analog-to-digital converter (ADC) resolution and a gain of 150.

### 2.4. Data Preprocessing

As a physiological electrical signal, an sEMG signal must be preprocessed before feature extraction [38]. The experimental signal processing steps we implemented are the ones that are commonly used in myoelectric gesture recognition research [35,39,40]. Due to the interference of external factors such as electrode displacement and channel damage during the experiment, the 64-channel high-density sEMG signals acquired may contain abnormal data channels. The HD-sEMG signals used in this study were obtained from arrayed electrode sheets. The distance between two adjacent electrodes was very close, and the surface EMG signals obtained were similar. Therefore, we were able to replace the sEMG signal of the abnormal channel using the mean value of the sEMG signals of the adjacent channels. First, we calculated the absolute maximum value (AMV) for each channel and set the median value of the AMV as the threshold. Second, the absolute value of each channel was compared to see if it exceeded the threshold. Finally, we replaced the sEMG signal of the abnormal channel with the average of two adjacent channels.

The frequency distribution of sEMG signals ranges between 10 and 500 Hz, but their main frequency range is 20–350 Hz [41,42]. During the acquisition of sEMG signals, the electrode shift caused by human body movement can cause low-frequency noise interference below 10 Hz. To remove the baseline drift caused by low-frequency noise and the interference caused by high-frequency noise in the sEMG signals, we used a fourth-order Butterworth band-pass filter at 20–350 Hz to filter the HD-sEMG signals. A 50 Hz power supply was used. To remove the industrial frequency interference, we used a 50 Hz notching filter.

An sEMG signal contains some background noise which it is difficult to separate. Wavelet functions in the time–frequency domain have better time–frequency characteristics, so in the effective signal in the noise-containing signal obtained through the wavelet transform, the energy is mainly concentrated in a small number of wavelet coefficients, so these coefficients are necessarily larger than the other coefficients in the wavelet transform domain. Contrary to the effective signal, the noise signal is generally uniformly dispersed throughout the time-domain space, and, correspondingly, its energy is also uniformly dispersed throughout the wavelet-domain space, coupled with the smaller amplitude of the noise, so the noise coefficients in the wavelet domain are also smaller. Therefore, the transformed wavelet coefficients can be grouped into two categories: one category is the noise coefficients, which are characterized by large numbers and small amplitudes; the other category is the signal coefficients, which are characterized by small numbers and large amplitudes. Furthermore, Stein’s Unbiased Risk Estimate (SURE) method selects the optimal threshold by evaluating the estimation error of the compressed signal. It does this by calculating a threshold value for each wavelet coefficient such that the mean square error of the reconstructed signal is minimized for a given signal-to-noise ratio. This process is adaptive and determines the most suitable threshold based on the characteristics of the signal and the noise level. Finally, the noise coefficients are filtered by the wavelet coefficient thresholds determined by the SURE method. Therefore, wavelet denoising was used in this study to further remove the background noise contained in the sEMG signal.

### 2.5. Methods

#### 2.5.1. HD-sEMG-Based Topographic Energy Maps

An HD-sEMG signal can measure the electrical activity of the muscle covering the restricted skin area and capture the feature distribution information of the sEMG signal in the time domain and the space domain of the whole muscle activity area. To visualize the dynamic energy distribution of muscle activity during different hand gestures, we introduced the normalized root mean square (NRMS) index to construct a series of topographic energy maps. The length of the sliding window has significant impacts on pattern recognition performance and on the real-time requirement of a less than 300 ms response time for prosthetic control [43]. Through continuous experiments, it was determined that employing a sliding window with a length of 200 ms achieves optimal performance in this study. Firstly, we calculated the root mean square (RMS) by computing the HD-sEMG signal of each channel through the sliding window. The RMS was calculated for each time window to obtain the average energy distribution as follows.

(1) $$R\left\{v\left[m\right]\right\}=\sqrt{\frac{1}{m}\sum_{i=1}^{m}v^{2}\left[i\right]}$$

where $R\left\{v\left[m\right]\right\}$ is the RMS value of the sEMG signals for each analysis window, $v\left[i\right]$ is the $i^{th}$ sample in the time window and m is the total number of windows.

Then, we normalized the RMS values across all channels by the maximum and minimum RMS. The normalized RMS values were symbolized by NR as follows.

(2) $$\mathrm{NR}\left(i\right)=\frac{R\left(i,j\right)-min\left(R\right)}{max\left(R\right)-min\left(R\right)}$$

where $NR\left(i\right)$ is the normalized RMS value of sEMG signals in the channel, $i,R\left(i,j\right)$ is the RMS value of channel i in the analysis window j, $min\left(R\right)$ is the minimum RMS value of channel i and $max\left(R\right)$ is the maximum RMS value of channel i.

#### 2.5.2. HD-sEMG-Based Recognition Algorithm

**Figure 2:**
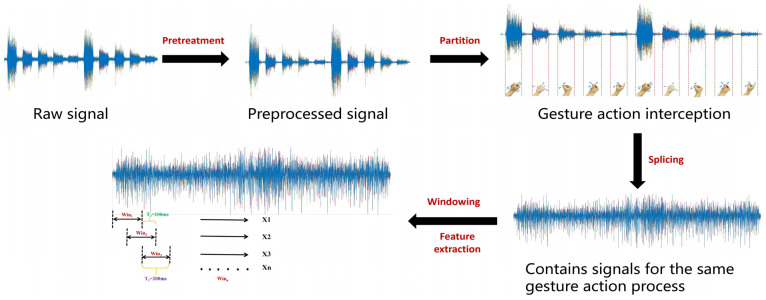
Flow diagram of gesture recognition.

Before feature extraction, the data for the same gesture need to be spliced and the resting EMG signals eliminated. Therefore, we used the start point detection algorithm to determine the onset points of different gestures. Firstly, we extracted the RMS values from the preprocessed EMG signals using a series of sliding windows. Secondly, based on the results of our multiple experiments, we used the average of the RMS values generated by all sliding windows as the threshold value. Finally, the sliding window corresponding to the RMS that exceeded the threshold was filtered out, and the start position of this window was used as the start point of each gesture action. Subsequently, to ensure that the data samples of each gesture action were balanced, we selected 5 s of data after the onset point of each gesture action as the endpoint, according to the design of the experimental paradigm (the sampling rate of the device was 2000 Hz, so the data length of each action was 5 × 2000). As shown in Figure 2, surface EMG signals containing the same gesture were manually spliced according to the onset point and the endpoint determined by the above method. After that, a dataset containing the same gesture repeated 20 times was obtained. Finally, the sEMG features were calculated using a series of sliding windows with a window length of 400 data points. The overlap between two adjacent windows was 50%.

##### Feature Extraction

To enhance the recognition rate, we needed to extract the effective sEMG features from the raw data before further classification. In general, we often extract the sEMG features from the time domain (TD), the frequency domain (FD) or the time–frequency domain (TFD). Among them, the features in TD are used most frequently in sEMG classification due to their easy implementation, low computational complexity and satisfactory performance [44]. FD analysis is also used to extract features, considering that it can present intuitively the frequency distribution of sEMG signals [44]. Moreover, TFD analysis has been developed as a useful tool for processing non-stationary bio-signals. Therefore, in this study, we extracted eight kinds of sEMG features, including the mean (MAV), the root mean (RMS), the variance (VAR), the waveform length (WL), the peak frequency (PKF), the mean frequency (MNF), the intermediate frequency (MDF) and the wavelet packet energy (WPE), from preprocessed sEMG signals to construct the original feature set for feature selection, and the calculation formulas for these indexes are shown in Table 1.

##### Feature Selection

Due to the variability of the same feature for different users, redundant features will cause an increase in computational cost and a decrease in pattern recognition. To address this issue, this study proposes an MPP algorithm combining mutual information, principal component analysis (PCA) and the Pearson correlation coefficient method to select the effective features.

In this study, based on the non-linear characteristics of sEMG signals, mutual information is selected to initially filter out the feature types with high relevance to the user and eliminate the feature types with low relevance to a specific user. Mutual information can be calculated by the following formulas:

(3) $$I_{i}(X_{i},Y_{i})=\sum_{x_{i}\in X}\sum_{y_{i}\in Y}p\left(x_{i},y_{i}\right)log\frac{p\left(x_{i},y_{i}\right)}{p\left(x_{i}\right)p\left(y_{i}\right)},i=1,2,\ldots ,N$$

(4) $$Mean_{MI}=\sum_{i=1}^{N}I_{i}(X_{i},Y_{i})∕N$$

where $x_{i}$ denotes the sEMG features extracted from channel i, $y_{i}$ denotes the label corresponding to the sEMG feature extracted from channel i, N is the number of channels, $Mean_{MI}$ denotes the average of the sEMG features’ mutual information for the N channels, $p\left(x,y\right)$ is the joint probability distribution function of variables X and Y, and $p\left(x\right)$ and $p\left(y\right)$ are the edge probability distribution functions.

The $Mean_{MI}$ indicates the level of relevance of each type of sEMG feature to a particular user. The higher the $Mean_{MI}$, the higher the relevance of this type of feature to the user. Although feature selection using MI can eliminate feature types with low relevance to a particular user and reduce feature dimensionality, the features extracted through the sliding window still produce a very high longitude (4990 × 64 dimensional feature vectors, where 4990 is the number of feature dimensions of the five gesture actions extracted through the sliding window and 64 is the number of channels). To ensure the generalization ability of the classifier, feature dimensionality reduction is necessary. In this study, we used PCA for feature dimensionality reduction. We used a 98% cumulative variance contribution as a threshold condition to determine the number of principal components for each feature type.

However, MI only considers the correlation between features and users and does not consider the similarity between different types of features. Therefore, we again use the Pearson correlation coefficient for feature selection to filter out the strongly correlated features that contain repetitive information. Finally, the optimal feature vectors that match a particular user are selected. The Pearson correlation coefficient can be calculated as follows.

(5) $$Pearson=\frac{\sum_{i=1}^{n}\left(x_{i}-\bar{x}\right)\left(y-\bar{y}\right)}{\sqrt{\sum_{i=1}^{n}{\left(x_{i}-\bar{x}\right)}^{2}}\sqrt{\sum_{i=1}^{n}{\left(y-\bar{y}\right)}^{2}}}$$

where $x_{i}$ and $y_{i}$ represent the ith features of different feature types, n represents the dimension of the feature vector, and $\bar{x}$ and $\bar{y}$ represent the means of different samples, respectively.

The MPP algorithm is shown in Algorithm 1.
**Algorithm 1** Mutual information–PCA–Pearson correlation coefficient (MPP)
**Input**: sEMG signal M
**Output**: Optimal feature vector B1**Action:**2Use the HD-sEMG data to initialize a feature set T={j|j=1,2,⋯,M}, where M represents the number of feature types.3Calculate the $Mean_{MI}$ for each feature in T by Equations (3) and (4).4The two EMG features with the lowest $Mean_{MI}$ values in T are eliminated, and the remaining features T are transferred to an empty set Φ.5Dimension reduction of features in Φ using PCA. Transfer the dimensionality-reduced features in Φ to the empty set ψ.6The principal component that contributes the most to the variance of each feature in ψ is used to form the feature vector A. 7The Pearson correlation coefficients between the vectors in A are calculated by using Equation (5).8Based on the results of step 7, four feature types with low relevance are filtered out from ψ. The features are fused to obtain the optimal feature vector $B=\left\{\psi_{1},\psi_{2},\psi_{3},\psi_{4}\right\}$ that matches a specific user.9**End**

##### Training and Evaluation

To ensure a fair comparison with other similar work, the same evaluation metrics were used for all experiments. During the model training phase, we used an 80–20 strategy to split the dataset: 80% of the data was used for the training set, and 20% was used for the test set. To reduce the effect of the time scale, it was necessary to normalize the training set before classification and transform the data with different specifications into a state with a mean of zero and a variance of one. To better improve the generalization ability of the model and reduce the risk of model overfitting, a five-fold cross-validation technique was used to train the model. A grid search method was introduced in this study to find the best parameters for each classifier. Moreover, classification accuracy is one of the most popular metrics. It is essential for the accurate realization of the user’s intent and directly presents the recognition results of the gesture task.

(6) $$Acc=\frac{Ncor}{Ntest}\times 100\%$$

where Acc is the classification accuracy, Ncor is the number of correctly classified samples and Ntest is the total number of tested samples.

## 3. Results

### 3.1. Time- and Frequency-Domain Results of Electromyographic Signals at Various Stages of Preprocessing

**Figure 3:**
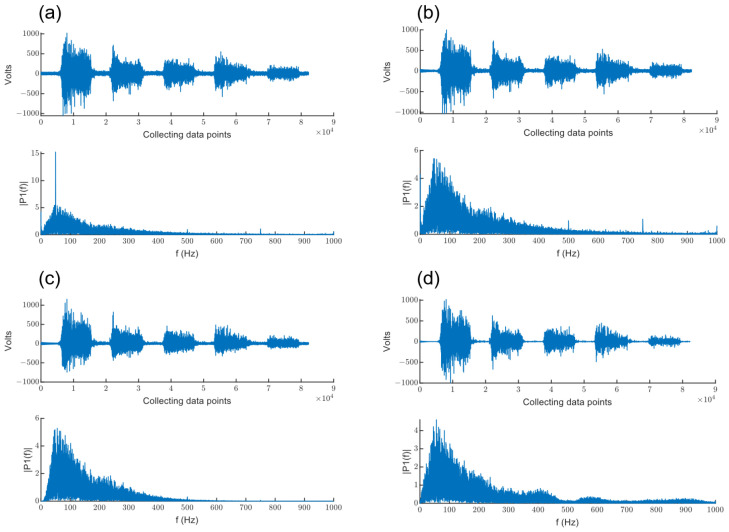
Display of preprocessing results at different stages. (**a**) Time-domain and frequency-domain figure of the original sEMG signal. (**b**) Time-domain and frequency-domain figure of the sEMG signal after the 50 Hz notching filter. (**c**) Time-domain and frequency-domain figure of the sEMG signal after the band-pass filter. (**d**) Time-domain and frequency-domain figure of the sEMG signal after wavelet denoising.

To show more intuitively the filtering effect of sEMG signals at each stage of the preprocessing process, Figure 3 shows the time-domain and frequency-domain results of the original sEMG signals after notching, band-pass filtering and wavelet denoising. From the time-domain figure in Figure 3a, we can see that there is a large baseline drift in the original sEMG signal, and from the frequency-domain figure, we can see that there is an industrial frequency interference of 50 HZ in the original sEMG signal. From Figure 3b,c, we can see that the baseline drift and industrial frequency interference of the sEMG signal have been effectively eliminated after the notch and band-pass filtering. From Figure 3d, it can be seen that the background noise contained in the sEMG signal is effectively removed after wavelet denoising.

### 3.2. Energy Activation Maps of Forearm Flexor Muscles for Different Gestures

**Figure 4:**
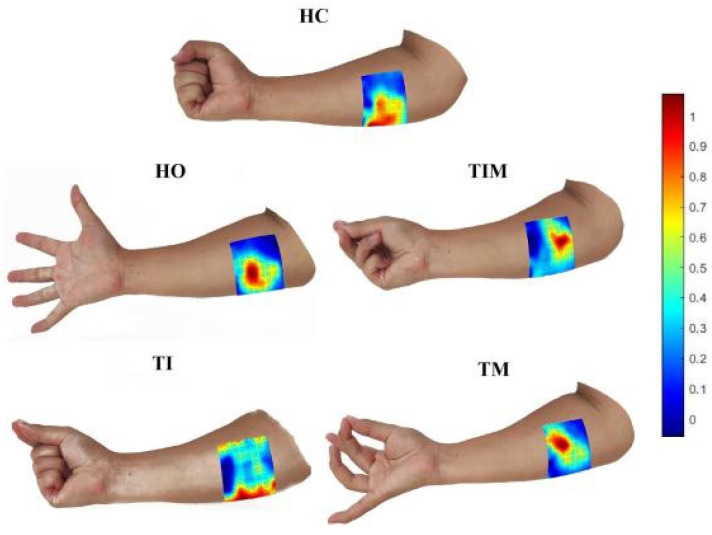
Energy activation of forearm flexor muscles for five gestures.

To visualize the muscle activity while executing different gestures, Figure 4 shows the topological energy of the five gestures based on HD-sEMG signals for a single user. As can be seen, the overall trend for both the hand open (HO) and hand closed (HC) tasks yields a larger range of sEMG activity and a larger area of energy map activation compared to other individual fingers (TI, TIM and TM). Secondly, when performing the movements of thumb and index fingers pinched (TI) and thumb, index and middle fingers pinched (TIM), there was large variability in muscle activation despite the similarity in the degree of execution of their movements.

### 3.3. Scatter Plots of HD-sEMG Features for Different Gestures

**Figure 5:**
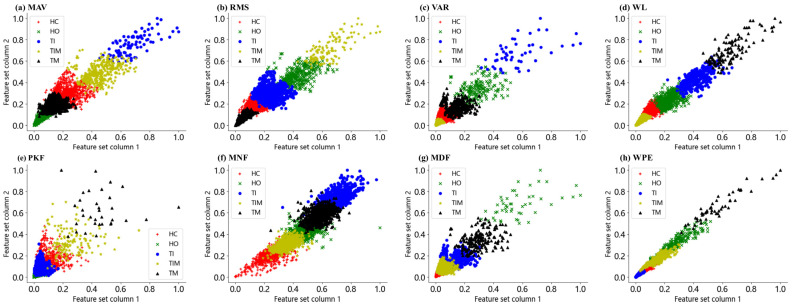
Scatter plots of different features corresponding to five gestures. (**a**) MAV. (**b**) RMS. (**c**) VAR. (**d**) WL. (**e**) PKF. (**f**) MNF. (**g**) MDF. (**h**) WPE.

To visualize the high-dimensional data as 2D images, we used PCA to dimensionality-reduce the features before performing K-means clustering. After PCA dimensionality reduction, we retained the two columns of features with the highest variance contribution. A K-means clustering model with five clusters was then created using K-means clustering and fitted to the dimensionality-reduced data. Finally, a two-dimensional scatterplot was presented to show the effect of clustering for different action modes. From Figure 5a–d, we can see that the distribution boundaries of MAV, RMS, VAR and WL features for different gesture tasks have a certain degree of differentiation, but for the hand open (HO) and hand closed (HC) motions there is still a large overlap in the features, which makes it difficult to achieve effective differentiation. Figure 5e–h show severe overlapping and difficult recognition for the five gestures, which demonstrates that it is difficult to distinguish these gestures based on single PKF, MNF, MDF or WPE features.

### 3.4. Feature Fusion for Different Gestures

**Figure 6:**
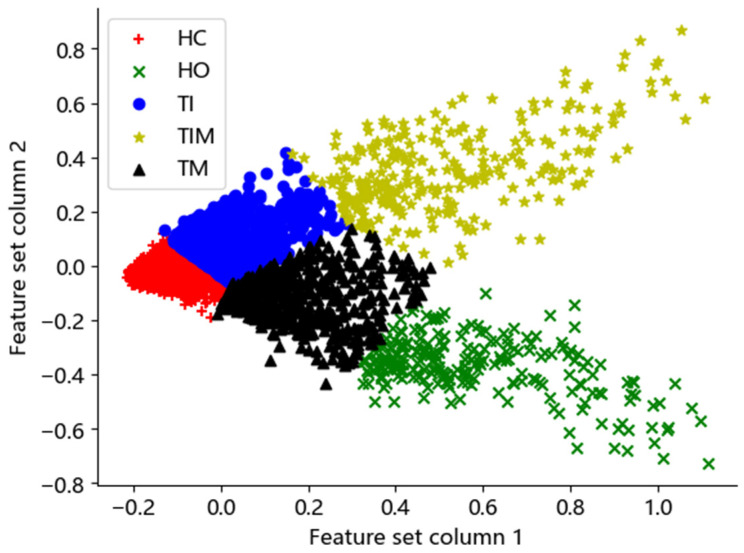
Scatter plot of the optimal features after feature selection.

To resolve the high overlap of feature distributions for single features in different gestures, we utilized the proposed MPP method to screen out the optimal features that matched specific users from the original feature set. After that, we used the K-means method to cluster them. Figure 6 shows the results for one subject. We can see that the optimal features have clear feature distribution boundaries and fewer overlapping parts for the five different gestures.

We used the MPP method to select the optimal features for other subjects. In Table 2, the features marked in red are the optimal features for each subject. We can see that the optimal subset of features constructed for each subject using MPP contains different types of EMG features. These differences also validate the problem of high variability among EMG signal users.

### 3.5. Comparison of Classifiers

To validate the superior performance of the MPP feature selection method proposed in this study when combined with the SVM model, we conducted a comprehensive comparison of the classification performance and training parameters across different models. For the KNN, SVM and AdaBoost machine learning models, we used the features selected through MPP as inputs, as shown in Table 3. In contrast, for the CNN and CNN_LSTM deep learning models, we utilized preprocessed raw sEMG signals as input data. It is worth noting that the parameters generated by machine learning models during training are only related to the number of features provided by the raw data, but deep learning models generate a large number of parameters due to continuous iterative training. As can be seen in Table 3, the iterative training of deep learning models generates a large number of parameters, which will greatly increase the computational cost. Although there is a slight difference in the average classification accuracy, SVM significantly decreases the model’s training time by approximately 95.67% compared to CNN_LSTM. Therefore, the gesture recognition framework that combines the optimal feature vectors obtained after MPP feature selection with the SVM classifier improves the pattern recognition efficiency compared to the deep learning model while ensuring classification accuracy.

### 3.6. Gesture Recognition Based on a Single Type of Feature

**Figure 7:**
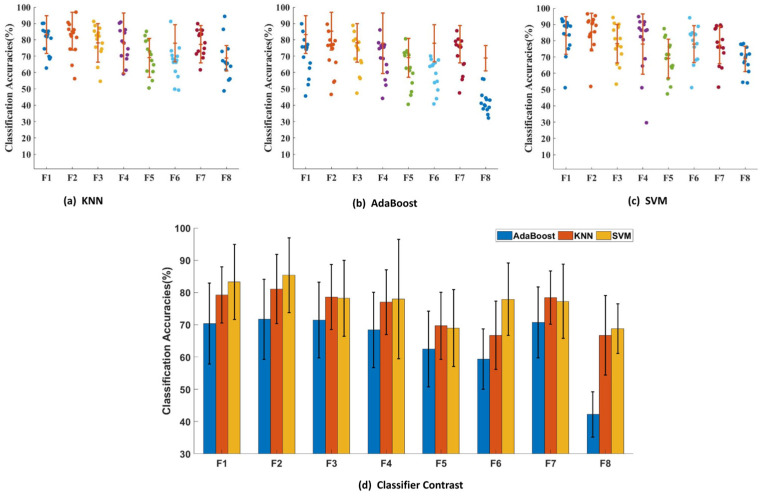
Average classification accuracy (%) of different gestures with different feature types for three classifiers: (**a**) KNN, (**b**) AdaBoost and (**c**) SVM. (**d**) Comparison of the eight features across the three classifiers.

To further investigate the advantages of feature fusion in gesture recognition, we first used a single feature type (MAV, RMS, VAR, WL, PKF, MNF, MDF and WPE) as the input to recognize the five gestures. We chose three types of classifiers, AdaBoost, KNN and SVM. Figure 7a–c show the classification of all subjects with eight features using the KNN, AdaBoost and SVM methods, respectively, and we can see that the classification effects of the same feature for different subjects varied greatly when using a single feature for classification. In Figure 7b, the recognition accuracies of the F1 and F2 features in the AdaBoost method fluctuate between 45% and 90% for different subjects. Further, we calculated and compared the average classification accuracies for individual features in Figure 7d. We found that the classification accuracy with AdaBoost classification was lower than 80%, only the classification accuracy of the RMS feature with the KNN classifier reached 81.1% and the classification accuracy with the SVM classifier was lower than 80%, except for MAV and RMS features.

### 3.7. Gesture Recognition Based on Fused Features

**Figure 8:**
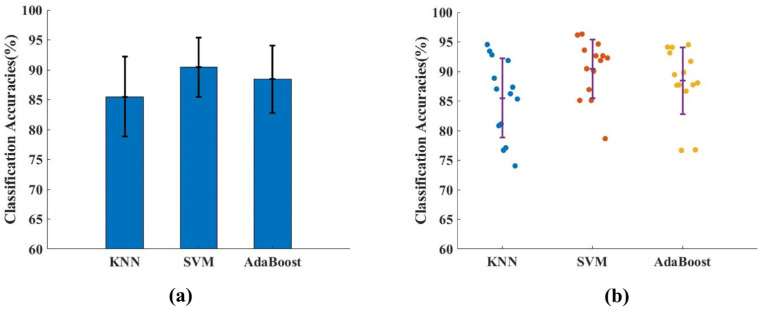
Feature fusion classification for the three classifiers. (**a**) Histogram of the average recognition accuracies for all subjects. (**b**) Scatter plot of the recognition accuracies for each subject.

Furthermore, we calculated and compared the classification accuracies of the three classifiers for the fused features. Figure 8a shows the average classification accuracy of the three classifiers, and Figure 8b shows the classification accuracy of the three classifiers for each participating subject. We can see that the average classification accuracy of fused features for all three classifiers is above 85%. In addition, compared with the other two classifiers, the SVM classifier is better, with an average classification accuracy of more than 91%.

To compare the classification performance of the four features selected in Table 2 that were highly correlated with specific user gestures and the fused features, we used SVM to classify them, and the recognition results are shown in Table 4. We can see that for the SVM classifier, the average classification accuracy for the fused features is above 91%, while the classification accuracy for the four original features is below 85%, which is an improvement of no less than 6% in classification accuracy.

**Figure 9:**
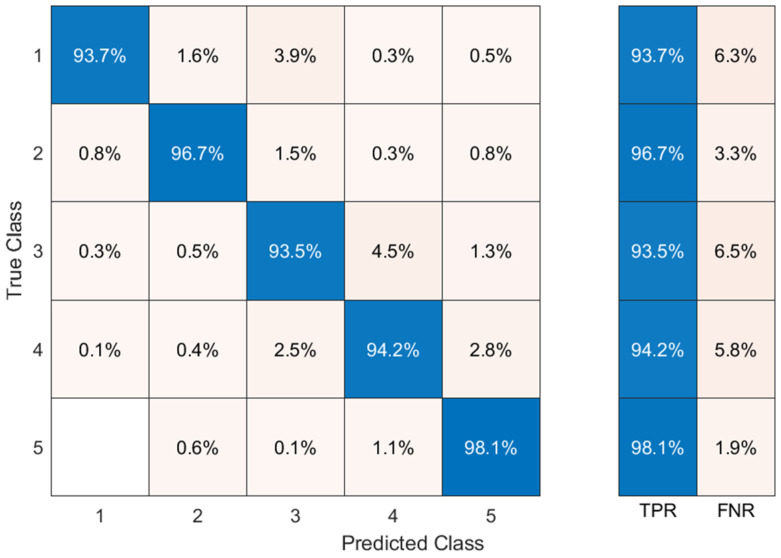
The classification accuracy (%) confusion matrix for the five gestures.

Figure 9 shows a confusion matrix of five different gesture actions for the SVM classifier with the fusion features of subject 3 as features. The rows of the matrix represent the actual gesture classes, and the columns represent the predicted classes. Therefore, the diagonal element is the correct classification rate and the non-diagonal element is the wrong classification rate, and it can be seen that the classification accuracy for all five gestures is above 92%.

### 3.8. Performance Comparison

**Figure 10:**
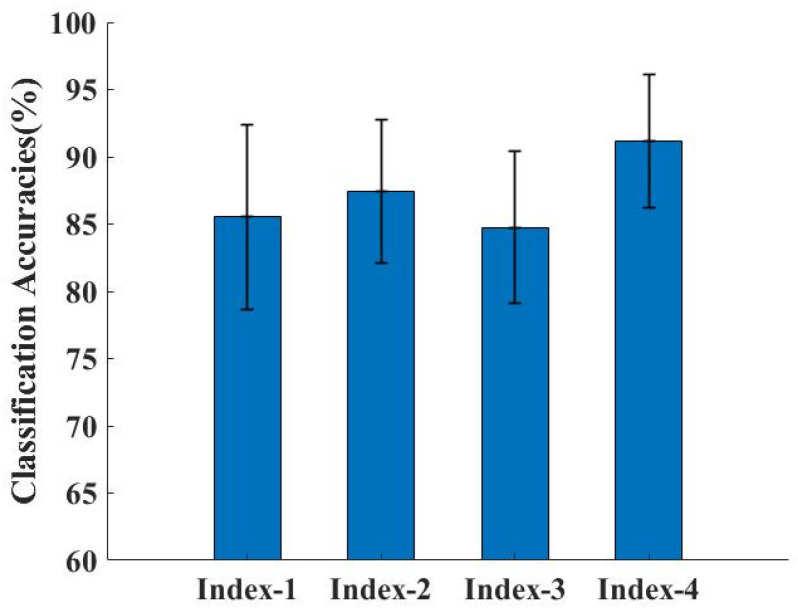
Average classification accuracies of different feature selection methods under the SVM classifier.

In Table 5, we compare the classification performance of three different feature selection algorithms combined with SVM. Our proposed MPP feature selection method combined with SVM classification achieved an accuracy of 91.16%. From the results for Index-1 and Index-2, we can see that using PCA to reduce the dimensionality of the feature subset after feature selection by MI can effectively improve the efficiency of pattern recognition. From the results of Index-2 and Index-4, it can be seen that after the initial feature selection using MI, the use of the Pearson correlation coefficient to further eliminate the features with strong correlations can effectively reduce the impact of feature redundancy on pattern recognition. In Figure 10, we can see that Index-4 is more robust and has a smaller standard deviation than the other three methods.

## 4. Discussion

sEMG-based gesture recognition has great potential to assist the interactive control of robotic devices, especially for restoring the function of the dexterous arm/hand [45]. In existing research, pattern recognition for sEMG signals is mainly categorized into machine learning and deep learning. In the process of actual human–computer interaction, we expect to obtain a plug-and-play model. However, deep learning requires a large amount of data in the training phase of the model, whereas machine learning has a unique advantage in this regard. Machine learning requires only a few features to train a model. The premise of ensuring a reliable machine learning classification model is to provide high-quality training features. In this respect, researchers have mainly focused on the development of new effective features and feature selection to improve the quality of features. However, in pattern recognition research based on sEMG signals, due to their dynamic nature and complexity, the same sEMG feature varies tremendously from one user to another even with the same gesture. Therefore, the user-specific EMG feature selection method becomes an effective way to solve this problem.

In previous studies, gesture recognition based on sEMG signals mostly employed sparse multi-channel sEMG, and the locations of these sensors were empirically selected without quantitative analysis [10]. However, an insufficient number of sEMG sensors can result in the absence of important muscle coverage and major myoelectric activity that is critical for gesture recognition. For example, placing the blue position in all the sEMG energy graphs (Figure 4) may ignore the large amplitude of muscle activity in the middle region, resulting in a decline in classification performance for gesture recognition. In this study, a 64-channel matrix high-density patch electrode was used to collect sEMG signals, which was sufficient to cover the muscle areas required for the five gestures described in this study and to ensure that no important information about muscle activity was missed.

This study aimed to use the good spatiotemporal features of HD-sEMG signals and form a sEMG feature set by extracting eight different types of sEMG features, namely, MAV, RMS, VAR, WL, PKF, MNF, MDF and WPE. Then, combined with the proposed feature selection algorithm, the optimal features suitable for specific users were selected and the feature dimensionality of the data was reduced, while the classification performance was taken into account, the computational cost was reduced and the classification accuracy was improved. Studies have shown that energy graphs calculated by HD-sEMG help visualize the dynamic energy distribution of muscle activity during gesture movement (Figure 4) and provide physiological cues for recognizing different gesture movements (Figure 5). From the energy diagram, it can be observed that the muscle activity intensity of the measured parts is different when the subjects make different gestures, which can also prove the feasibility of using pattern recognition to complete the classification of gestures.

Excellent features play a crucial role in the classification results of a model. In machine learning, methods for feature selection are categorized into three categories: filter, wrapper, and embedding methods. In existing studies, only one of the above three categories is usually used for selection. In this study, based on the non-linear characteristics of sEMG signals, mutual information was selected to initially filter out the feature types with high relevance to the user and eliminate the feature types with low relevance to a specific user. Although the MI feature selection can filter out the appropriate feature types for a specific user, the feature extraction through the sliding window still produces a very high latitude (4990 × 64 dimensional feature vectors; each channel produces 4990 eigenvalues out of 64 channels) In this case, to ensure the generalization ability of the classifier, it is necessary to carry out feature dimensionality reduction. Therefore, in this study, PCA was used to reduce the dimensionality of the filtered features, and, at the same time, the Pearson correlation coefficient was used to calculate the correlation between different features after dimensionality reduction. The impact of redundant features on pattern recognition performance was effectively reduced by further eliminating feature types with high correlations. After obtaining the feature information that matches a specific user, the machine learning model can achieve high classification performance. Compared with the huge amount of data required for deep learning model training, this study can ensure recognition performance while reducing the computational cost and user burden. Secondly, using the feature engineering method proposed in this study provides a solution to the problem of degraded recognition performance of cross-user gesture recognition patterns. As can be seen from Figure 7, when a single feature is used for classification, the average classification accuracy of 14 subjects in the three classifiers is about 80%, and the classification accuracy of eight original features is lower than 80% when the AdaBoost classifier is used for classification. When the KNN classifier is used to complete the classification, only RMS classification accuracy reaches 81.1%. When the SVM classifier is used to complete the classification, except for MAV and RMS, the classification accuracy for other features for the five gestures is below 80%. However, as can be seen in Figure 7, when the features determined using the feature selection method proposed in this study are used for classification, the average classification accuracy is 91%, which proves the effectiveness of the MPP method.

When examining the selected feature types, it was found that even when using the same feature selection method, there were differences in the feature types selected by 14 subjects (Table 2), which was mainly due to differences in the subjects (such as muscle strength, daily activities, muscle position, etc.). This confirms that we previously designed or performed in a user-specific way in EMG pattern recognition.

Finally, there are some limitations to this study. We acknowledge that the proposed feature selection methods based on MPP may generate relatively higher computational complexity than traditional TD feature sets. In addition, the EMG signals of only 14 subjects were collected in this study. To further validate the robustness of the method, the expansion of the dataset is necessary in future work. Nevertheless, other publications have published results based on similar numbers of test persons. Rojas-Martínez et al. collected EMG signals from 12 healthy subjects to study patterns of sEMG spatial distribution over upper limb muscles during voluntary isometric contractions [46]. C. Amma. et al. collected EMG signals from five healthy subjects to study a gesture classification task [47]. Zhang. et al. collected EMG signals from 15 stroke patients to interpret the patients’ motor intentions [16]. Meanwhile, different gesture movements only require muscles in specific areas for control. Compared with acquiring sparse multi-channel surface EMG signals, HD surface EMG signals will generate more redundant channels for surface EMG pattern recognition analysis. Channel selection methods can reduce the number of channels while ensuring that no information related to the task state is lost. Therefore, combining channel selection with feature selection in future work could further improve the performance of pattern recognition.

## 5. Conclusions

In this study, we used HD-sEMG signals to recognize five hand gestures at specific muscle sites. The energy activation diagrams calculated from HD-sEMG signals can help visualize the dynamic energy distribution of muscle activity in different parts during different gestures and verify the feasibility of recognition. By using the feature selection algorithm proposed in this study, the sEMG data of 14 healthy human subjects were processed. Compared with the traditional classification effect of single sEMG features, this method can effectively identify a small number of suitable sEMG features and fuse them to form the optimal features suitable for specific users, significantly improving the performance of HD-sEMG gesture recognition and classification. The research provides a practical approach for cross-user gesture recognition.

## Figures and Tables

**Table 1:** Eight myoelectric features and mathematical definitions.

Feature	Mathematical Expression
Mean Absolute Value (MAV)	$MAV=\frac{1}{N}\sum_{i=1}^{N}\left|x_{i}\right|$
Root Mean Square (RMS)	$RMS=\sqrt{\frac{1}{N}\sum_{i=1}^{N}x_{i}^{2}}$
Variance (VAR)	$VAR=\frac{1}{N-1}\sum_{i=1}^{N}x_{i}^{2}$
Waveform Length (WL)	$WL=\sum_{i=1}^{N-1}\left|x_{i+1}-x_{i}\right|$
Peak Frequency (PKF)	$PKF=max\left(p_{j}\right),j=1,\ldots ,M$
Mean Frequency (MNF)	$MNF=\sum_{j=1}^{M}f_{j}p_{j}/\sum_{j=1}^{M}p_{j}$
Median Frequency (MDF)	$MDF=\frac{1}{2}\sum_{j=1}^{M}p_{j}$
Wavelet Packet Energy (WPE)	$WPE=\sum_{i=0}^{N-1}x^{2}\left[n\right]$

Note: $x_{i}$ represents the EMG signal in a segment i, N  represents the length of the EMG signal, $f_{j}$ is the frequency of the spectrum at frequency bin j, $p_{j}$ is the EMG power spectrum at frequency bin j and M is length of the frequency bin.

**Table 2:** Feature categories of 14 screened subjects.

Subject	Feature Selection
1	(**MAV**, RMS, VAR, **WL**, PKF, **MNF**, **MDF**, WPE)
2	(**MAV**, RMS, **VAR**, **WL**, PKF, MNF, **MDF**, WPE)
3	(MAV, **RMS**, VAR, **WL**, **PKF**, MNF, **MDF**, WPE)
4	(MAV, **RMS**, VAR, **WL**, PKF, **MNF**, **MDF**, WPE)
5	(**MAV**, RMS, **VAR**, **WL**, PKF, MNF, **MDF**, WPE)
6	(MAV, RMS, **VAR**, **WL**, **PKF**, MNF, MDF, **WPE**)
7	(MAV, **RMS**, **VAR**, **WL**, PKF, **MNF**, MDF, WPE)
8	(MAV, **RMS**, VAR, **WL**, PKF, **MNF**, MDF, **WPE**)
9	(MAV, **RMS**, VAR, **WL**, PKF, **MNF**, **MDF**, WPE)
10	(**MAV**, RMS, **VAR**, **WL**, PKF, MNF, **MDF**, WPE)
11	(MAV, **RMS**, VAR, **WL**, PKF, **MNF**, **MDF**, WPE)
12	(**MAV**, **RMS**, **VAR**, WL, **PKF**, MNF, MDF, WPE)
13	(MAV, **RMS**, **VAR**, **WL**, PKF, MNF, **MDF**, WPE)
14	(MAV, **RMS**, VAR, WL, **PKF**, MNF, **MDF**, **WPE**)

**Table 3:** Comparison of performance parameters of different methods.

Methods	Parameters	Time (s)	Acc (%)
KNN	✗	136.14	85.51
AdaBoost	✗	45.15	88.43
SVM	✗	46.11	91.16
CNN	166,789	991.728	90.78
CNN_LSTM	241,029	1063.68	92.43

✗ indicates that the machine learning classification model does not generate additional training parameters.

**Table 4:** Comparison of SVM classification accuracy between fused features and individual features.

Subject	Feature Fusion	Feature Selection			
		F1	F2	F3	F4
1	96.15	93.35	94.79	94.07	87.26
2	95.11	91.75	86.54	87.74	87.74
3	96.31	92.15	91.59	83.33	89.26
4	93.59	82.45	82.45	51.2	79.09
5	90.46	89.18	75.03	86.46	76.12
6	86.92	51.14	51.14	80.25	51.47
7	85.11	74.44	65.63	64.47	64.98
8	90.06	90.46	80.62	76.12	75.96
9	92.63	91.02	89.82	84.05	89.61
10	94.63	89.58	90.14	91.58	88.78
11	91.83	77.64	68.91	68.19	63.30
12	92.63	83.57	85.49	81.33	64.18
13	78.64	95.35	76.28	79.65	75.56
14	92.26	89.42	86.62	88.78	72.52
**Mean (%)**	**91.17**	**85.11**	**80.36**	**79.80**	**79.13**
**Std**	4.95	11.49	12.01	11.65	11.88

**Table 5:** Average classification accuracies of different feature selection methods under the SVM classifier.

Index	Methods	Average Acc (%)
Index-1	Yan et al. [34].	85.51
Index-2	Khushaba et al. [35]	87.43
Index-3	Angari et al. [36]	84.74
Index-4	Our work	91.16

## Data Availability

The data presented in this study are available upon request from the first author.
